# A New Species of the Genus *Scincella* Mittleman, 1950 (Squamata: Scincidae) from the Hengduan Mountains, Sichuan Province, Western China

**DOI:** 10.3390/ani16040592

**Published:** 2026-02-13

**Authors:** Fan Liu, Hongli Pu, Songwen Tan, Jiahao Chen, Bing Lyu, Guocheng Shu, Yayong Wu, Bingjun Dong, Peng Guo

**Affiliations:** 1 College of Life Sciences, Shenyang Normal University, Shenyang 110034, China; liufan22124@163.com (F.L.);; 2 Faculty of Agriculture, Forestry and Food Engineering, Yibin University, Yibin 644005, China; 3 College of Life Sciences, Xinjiang Agricultural University, Urumqi 830052, China

**Keywords:** Hengduan mountains, phylogenetic taxonomy, morphological comparison, lizard

## Abstract

The genus *Scincella* Mittleman, 1950 of the family Scincidae currently includes 50 species. However, due to challenges in sample collection, the taxonomy and phylogeny of this genus remain poorly resolved. This study conducted a taxonomic study of the genus *Scincella* based on three mitochondrial gene fragments (12S, 16S, COI) combined with morphological comparisons. The results indicated the specimens collected in Heishui County, Sichuan Province, China, represented a previously undescribed species. This new taxon is formally described as *Scincella heishuiensis* **sp. nov**. Phylogenetically, the new species is most closely related to *S. wangyuezhaoi*, which is another species recently described in Wenchuan County, Aba Prefecture, Sichuan Province. As a new species, the uncorrected genetic distance between this new species and its closest relative within the genus, as calculated based on 16S rRNA, is 5.45%. The new species can be distinguished from other closely related species by the following morphological characters: (1) 4 dorsal scale rows between dorsolateral stripes; (2) 5–7 superciliary; (3) 28 midbody scale-rows; (4) 24–25 gulars; (5) during the breeding season, the ventral scales and preanal exhibit a reddish-brown color; and (6) a black lateral stripe extends from behind the eye to the posterior third of the tail near the cloaca. In the phylogenetic analysis, the new species and *S. wangyuezhaoi* form an independent lineage, distinctly separate from all other taxa included in the study. The new species has currently been recorded and discovered in Heishui County in Sichuan Province, China. This description thus increases the number of members in the genus *Scincella* in China to 17 species.

## Simple Summary

The skink genus *Scincella*, a member of the family Scincidae, is characterized by a wide distribution and conservative morphology. This study integrates molecular and morphological data to conduct phylogenetic analyses and comparative morphology of *Scincella* population in the Hengduan Mountains. Our findings include the discovery of a new species and the clarification of its phylogenetic relationships within the genus. Significant morphological differences were observed between specimens collected from Heishui County and other known *Scincella* species. When combined with phylogenetic analysis results, these specimens were identified as a new species, formally described as *Scincella heishuiensis* **sp. nov**. Phylogenetic reconstruction reveals that *Scincella heishuiensis* **sp. nov**. forms a sister group with *S. wangyuezhaoi* while exhibiting distinct morphological differentiation from all other *Scincella* species. This study not only enriches the diversity of the genus *Scincella* but also provides new insights into its phylogeny history and evolutionary patterns. The results indicate that the diversity of *Scincella* in the Hengduan Mountains is underestimated and requires further research and exploration.

## 1. Introduction

The Hengduan Mountains (HDM), located on the eastern Qinghai–Xizang Plateau (QXP), are characterized by dramatic elevational gradients that create distinct vertical ecological zones, harboring exceptionally high biodiversity. This region has been recognized as the original and evolutionary center for several taxonomic groups [1]. Recent taxonomic investigations have revealed numerous new species in the area, including snakes [2,3,4], lizards [5,6], and frogs [7]. The increasing description of new taxa in this region indicates a significant underestimation of its biodiversity [8].

The genus *Scincella* Mittleman, 1950, which belongs to the family Scincidae, was originally described based on the type species *S. lateralis*. Wang et al. divided Chinese species into three groups based on morphological comparisons and recognized 14 species in China [8]. Che et al. moved three *Scincella* species inhabiting Xizang, China to *Asymblepharus* [9], which was established by Eremtschenko [10], and would later be treated as a subjective junior synonym of *Ablepharus* [11]. In recent years, several new species have been described sequentially in Western China [5,6,12]. Currently, the genus *Scincella* comprises 50 species and exhibits a broad distribution across East Asia, Southeast Asia, and North America [13].

In China, the HDM represents the primary distribution area for *Scincella* species, with 10 species recorded in this region: *S. schmidti*, *S. liangshanensis*, *S. wangyuezhaoi*, *S. potanini*, *S. monticola*, *S. tsinlingensis*, *S. modesta*, *S. doriae*, *S. barbouri* and *S. reevesii* [5]. However, recent studies have identified several potential taxonomic issues [5,6], indicating the possibility of the presence of a substantial number of cryptic species in the area.

During recent field surveys in the HDM, we collected several *Scincella* specimens, including a distinctive population from Heishui County, Sichuan, China. Morphological analyses revealed significant differences from congeners, which were further supported by molecular phylogenetic evidence showing substantial divergence from known species. Based on integrated morphological and molecular phylogenetic analyses, we formally describe these specimens as a new species. Subsequent phylogenetic analysis based on three mitochondrial DNA genes (COI, 12S rRNA, and 16S rRNA) confirmed that this population from Heishui County, Sichuan Province, belongs to the genus *Scincella*. The analysis also revealed that it forms a distinct sister lineage to *S. wangyuezhaoi* from Wenchuan County, Sichuan Province. Further examination of the scalation, coloration, and morphology of the Heishui population, along with comparisons to other species, demonstrated clear morphological distinctions from both its sister species and existing species within the genus *Scincella*. Therefore, we describe the skinks from Heishui County in the northern HDM of China as a new species of *Scincella*.

## 2. Materials and Methods

**Figure 1:**
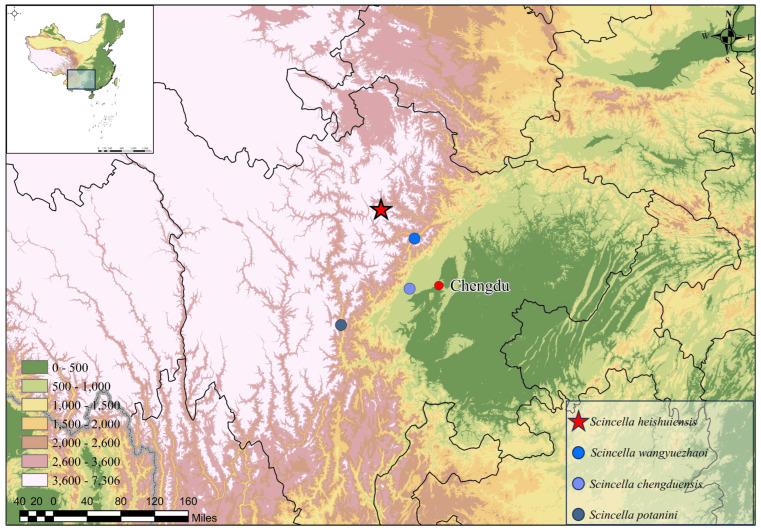
A map showing the type localities of the genus *Scincella* species in the Hengduan Mountains region of southwestern China. *Scincella heishuiensis* in Heishui County, Aba Prefecture, Sichuan Province; *S. wangyuezhaoi* in Wenchuan County, Aba Prefecture, Sichuan Province; *S. chengduensis* in Chengdu City, Sichuan Province; and *S. potanini* in Kangding City, Garzê Prefecture, Sichuan Province. Elevation data sourced from the Geospatial Data Cloud (2024).

**Specimen collection.** The specimens of *Scincella* sp. were collected from Heishui County, Sichuan, China (Figure 1). After the livers were taken, the specimens were fixed in a 10% formaldehyde solution for three days and then transferred to 80% ethanol for long-term preservation. The liver samples were kept at 85% ethanol in −20°. All specimens and samples are deposited at Yibin University (YBU).

### 2.1. Morphology Analyses

The two unidentified specimens were morphologically examined and recorded following Zhao et al. and Nguyen et al. [14,15,16,17,18]. Body dimensions were taken with a digital caliper to the nearest 1 mm. Symmetric mensural head characters were taken on the right side of the head only, unless this was damaged, in which case they were done on the left. Meristic characters were recorded on both sides, and the average was used in the analysis. All data were collected by the senior author to avoid inter-observer bias [18,19].

The characters measured and recorded and their abbreviations are as follows: snout–vent length (SVL, distance from tip of snout to posterior edge of vent); tail length (TAL, distance from posterior margin of vent to tip of tail); axilla–groin distance (AGD, distance between posterior edge of forelimb insertion and anterior edge of hindlimb insertion); forelimb length (FLL, measured from forelimb insertion to tip of finger IV or longest digit); hind-limb length (HLL, measured from hind-limb insertion to tip of toe IV or longest digit); toe IV length (T4L, measured from the most basal part to tip of toe IV); finger IV length (F4L, measured from the most basal part to tip of finger IV); midbody scale-row count (MBSR, number of longitudinal scale rows measured around widest point of midbody); dorsal scale rows between dorsolateral stripes (DBR, number of dorsal scale rows at midbody between dark dorsolateral stripes, following Inger; enlarged, differentiated nuchal count (NU, X pairs or absence); paravertebral scale-row count (PVSR, number of scale rows counted between parietals and the just posterior margin of hindlimbs); ventral scale-row count (VSR, number of scale rows counted between gulars and precloacal); superciliary count (SC, left/right); supralabial count (SL, left/right); infralabial count (IfL, left/right); superciliary count (SC, left/right); supraocular count (SO, left/right); enlarged temporal count (TEM, left/right); number of enlarged, undivided lamellae beneath finger IV (F4S, left/right); number of enlarged, undivided lamellae beneath toe IV (T4S, left/right); frontoparietal in contact with each other (FP); prefrontal in contact with each other (PF); chin-shields: paired large scales behind mental or postmentals; gulars: many minor scales in the center of the ventral head (number of scale rows counted between the first scale behind the chin-shields and the middle of the forelimb); appearance of dorsolateral stripes (ADLS).

### 2.2. Molecular Analyses

Total DNA was extracted from the liver tissue of two samples using the M5 HiPer Universal DNA Mini Kit (Mei5 Biotechnology Co., Ltd., Beijing, China). Three mitochondrial DNA fragments were amplified, 12S ribosomal RNA (12S) using primers L1091-F and H1478-R (5′–3′: AAACTGGGATTAGATACCCCACTAT/GAGGGTGACGGGCGGTGTGT), 16S ribosomal RNA (16S) using primers 16SL-F and 16SL-R (5′–3′: TGTTTACCAAAAACATAGCCTTTAGC/TAGATAGAAACCGACCTGGATT), cytochrome c oxidase subunit I (COI) using primers Chmf4 and Chmr4 (5′–3′: TYTCWACWAAYCAYAAAGAYATCGG/ACYTCRGGRTGRCCRAARAATCA) [20]. PCR cycling parameters followed the original protocols from these studies. PCR products were purified using commercial kits prior to sequencing. Bidirectional sequencing was performed by Sangon Biotech (Shanghai, China). Phylogenetic analyses incorporated 21 *Scincella* species and one outgroup (*Sphenomorphus cryptotis*) [21], with sequences retrieved from GenBank (Table 1). Sequence editing was conducted in Lasergene v15.1, followed by Clustal W alignment and quality verification in MEGA v11.0 [22]. Raw nucleotide sequences were manually validated using SeqMan v.7.1.0.44 [23].

Prior to phylogenetic analyses, the optimal substitution model for each gene partition was selected using PartitionFinder 2.1.1 [24] under the Akaike Information Criterion. Phylogenetic trees were constructed using Maximum Likelihood (ML) and Bayesian Inference (BI) methods. BI analyses were conducted in MrBayes 3.2.2 [25], using three heated chains and one cold chain, with three independent runs of 10 million generations each (sampling every 2000 generations). The initial 25% of samples were discarded as burn-in, achieving a potential scale reduction factor (PSRF) < 0.005 across all runs. ML analyses were performed in IQ-TREE [26] with 1000 standard bootstrap replicates (BS). Bayesian posterior probabilities (BS) ≥ 0.95 and PP ≥ 95 are considered to indicate strong support for tree nodes [27]. Phylogenetic trees were visualized and annotated in FigTree 1.4.3 [28]. Given the difficulties in collecting samples of Scincidae species, coupled with the numerous discoveries of new species within the genus *Scincella* in recent years, and considering that this specimen morphologically most closely resembles the recently described *S. wangyuezhaoi*, this study exclusively selected species from the genus *Scincella* to construct the phylogenetic tree.

The uncorrected *p*-distance between species was calculated for 16S and COI sequences in MEGA v11.0 with 1000 bootstrap replications [22] respectively.

## 3. Results

### 3.1. Morphology Examination

The newly collected specimens exhibit morphological affinities with *Scincella* by sharing key scalation features, including well-developed eyelids, absence of supranasal, and one pair of preanal. Meanwhile, they can be distinguished from congeners by the following unique characters: four dorsal scale rows between dark dorsolateral stripes at midbody, specific midbody-scale-row count, presence or absence pattern of postnasal scales, and loreal scale number.

### 3.2. Molecular Phylogeny

The final aligned dataset consists of 1462 nucleotide positions (490 bp from 16S RNA, 353 bp from 12S RNA, 619 bp from COI). The best-fit nucleotide substitution model (GTR + I + G) was determined for the combined dataset. All novel sequences generated here have been deposited in GenBank (Table 1).

**Figure 2:**
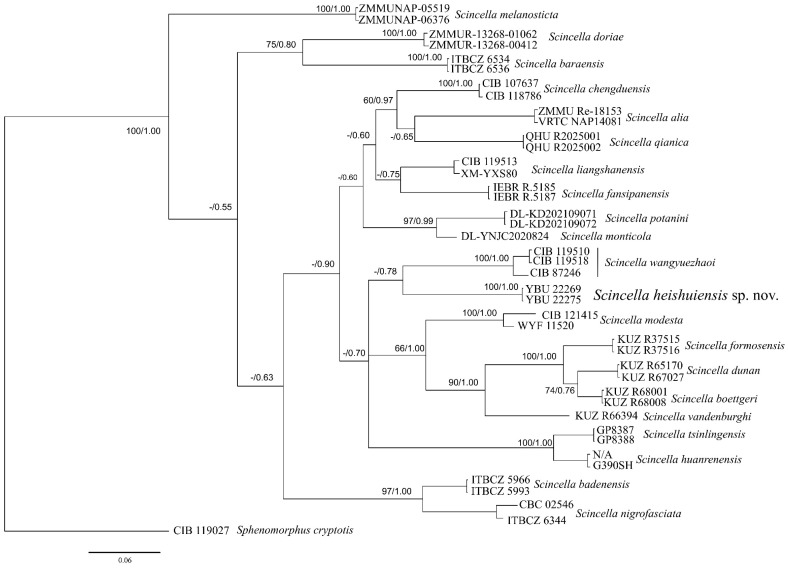
Phylogenetic tree reconstructed based on three mitochondrial fragments, ultrafast bootstrap supports (BSs) from ML analyses and Bayesian posterior probabilities (PPs) from BI analyses were shown above branches (BS/PP). Those lower than 50 are displayed as “-”.

Both BI and ML analyses yielded largely consistent phylogenetic topologies, with minor discrepancies in node support values observed at certain positions (Figure 2). All recognized *Scincella* species, including the two newly collected specimens, formed a strongly supported monophyletic group (100 BS and 1.0 PP). The new specimens constituted a distinct clade with high support indices (BS = 100, PP = 0.99), exhibiting a sister-group relationship with the clade containing three *S. wangyuezhaoi* individuals.

Based on 16S rRNA sequences, interspecific genetic distances among *Scincella* species ranged from 2.19% (*S. tsinlingensis* vs. *S. huanrenensis*) to 10.18% (*S. huanrenensis* vs. *S. gemmingeri*). Based on COI sequences, the distances ranged from 7.6% (*S. dunan* vs. *S. boettgeri*) to 23.1% (*S. melanosticta* vs. *S. vandenburghi*). The newly proposed taxon (comprising the two novel samples) exhibited genetic distances of 5.45% (16S) and 15.2% (COI) relative to *S. wangyuezhaoi* (Table 2 and Table 3).

Integrating morphological comparison and molecular phylogeny, we confirm that the specimens collected from Heishui County, Sichuan Province, China, represent a distinct species. We formally describe it herein.

### 3.3. Taxonomic Account

***Scincella heishuiensis* sp. nov**.

**Figure 3:**
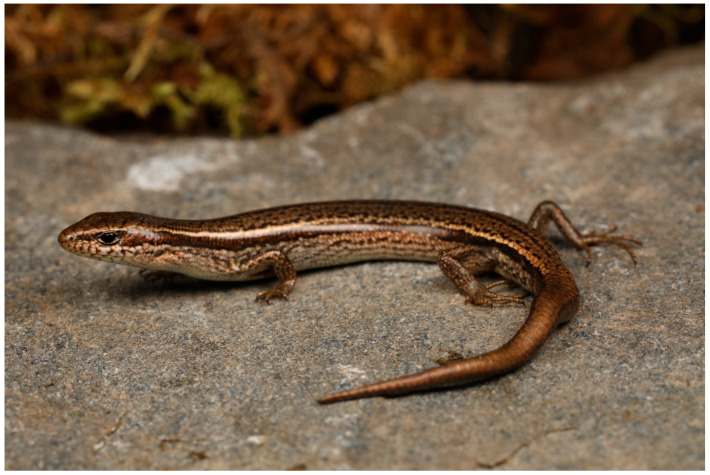
General view of the holotype of *Scincella heishuiensis* sp. nov. (YBU 22269) in life.

**Figure 4:**
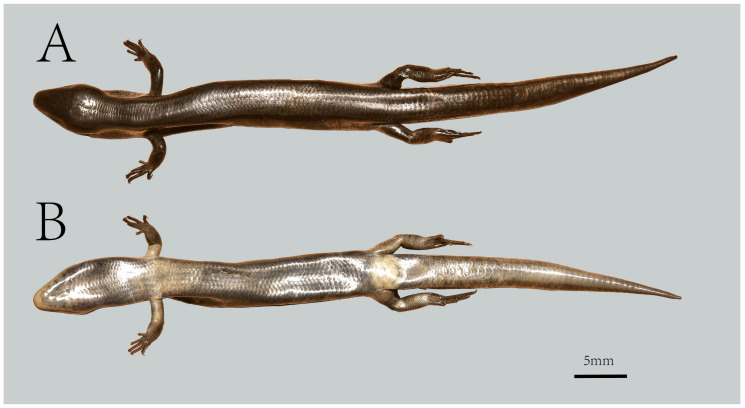
Dorsal (**A**) and ventral (**B**) views of the holotype of *Scincella heishuiensis* sp. nov. (YBU 22269) in preservative.

**Figure 5:**
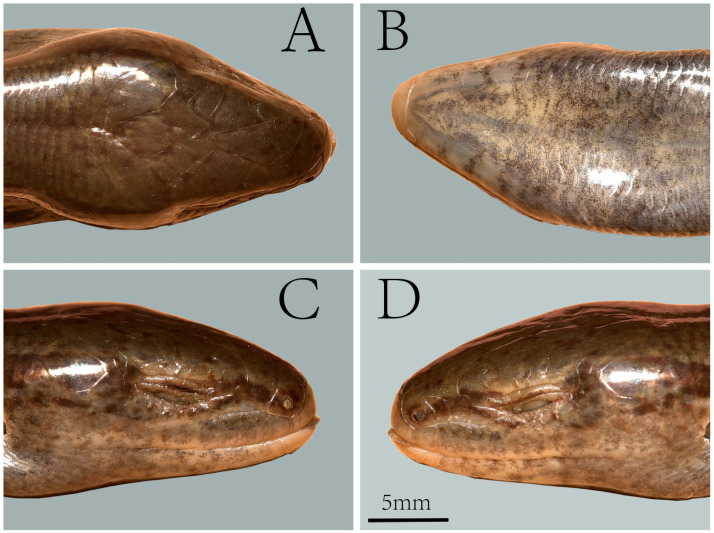
Dorsal (**A**), ventral (**B**), right lateral (**C**), and left lateral (**D**) views of the head of the holotype of *Scincella heishuiensis* sp. nov. (YBU 22269).

**Figure 6:**
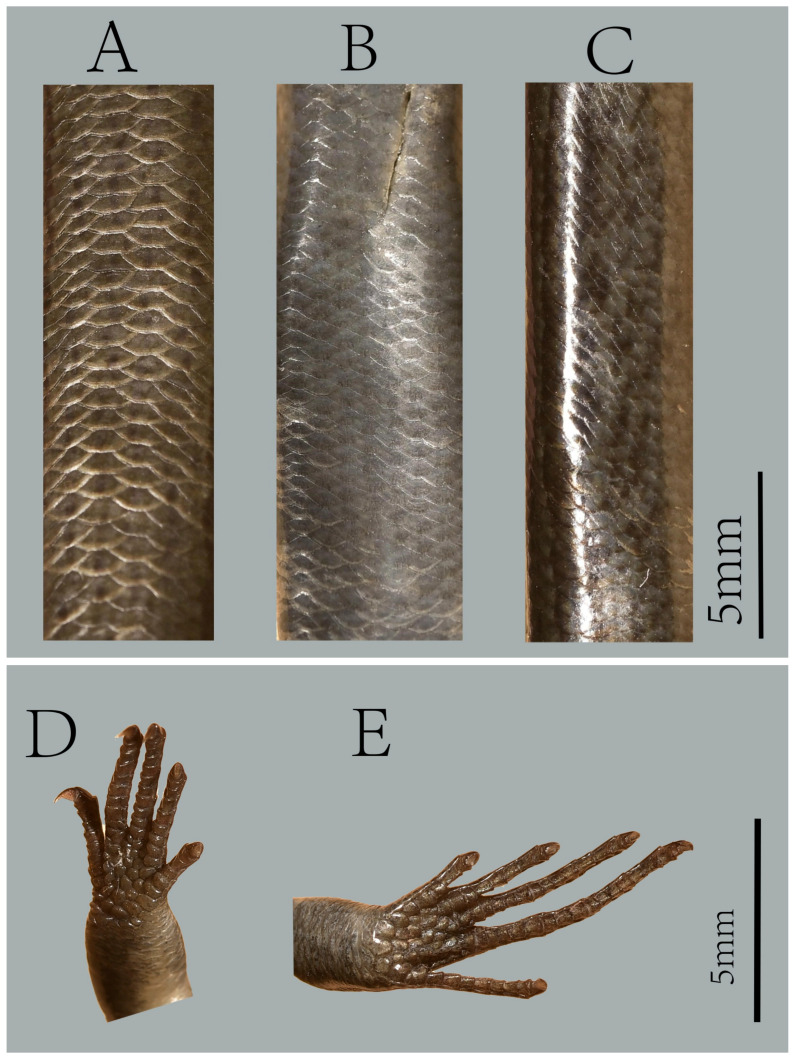
Holotype of *Scincella heishuiensis* **sp. nov**. (YBU 22269). Dorsal (**A**), ventral (**B**), and lateral (**C**) views of body; ventral views of hand (**D**) and foot (**E**).

Figure 3, Figure 4, Figure 5 and Figure 6.

*Scincella wangyuezhaoi* Jia, Ren, Jiang & Li, 2023 [5].

**Holotype.** YBU 22269, an adult male, collected from Mt. San’ao, Heishui County, Aba Tibetan and Qiang Autonomous Prefecture, Sichuan Province, China (32.02° N, 102.92° E, 2900 m a.s.l), in June 2022.

**Paratype (n = 1).** YBU 22275, an adult male, the same locality and date as the holotype.

**Diagnosis.** *Scincella heishuiensis* **sp. nov**. exhibits the following characteristics which can distinguish from its congeners: (1) four dorsal scale rows between dorsolateral stripes; (2) 5–7 superciliary; (3) 28 midbody scale rows; (4) 24–25 gulars; (5) during the breeding season, the ventral scales and preanal exhibit a reddish-brown color; and (6) a black lateral stripe extends from behind the eye to the posterior third of the tail near the cloaca.

**Description of the holotype.** YBU 22269, an adult male. The body is slender and of medium size, with a snout–vent length (SVL) of 40.90 mm, and TAL of 39.25 mm. The neck is indistinct from the head. The axilla–groin distance (AGD) 29.65 mm, (AGD/SVL 0.72). The tail is short and thick, with a length (TAL) of 39.25 mm (TAL/SVL 0.96). The forelimb length (FLL) is 8.20 mm, and the hind limb length (HLL) is 12.87 mm. Forelimb digit lengths: IV > III > II > V > I; Hindlimb digit lengths: IV > III > II > V > I. Fingers are slender, F4L 2.58 mm, T4L 4.96 mm. Subdigital lamellae single, arranged in an overlapping series: F4S 8/8, T4S 13/13.

The rostral is blunt and rounded. The supranasal is absent. Two prefrontals, rhomboid-shaped, separated by the frontonasal, with the two scales contacting only at one point. Two frontoparietals, irregularly pentagonal, adjacent and in contact with each other. One parietal, the largest on the dorsal head, pentagonal in shape, with a width approximately two-thirds of its length; its lateral margins contact only two supraoculars and one prefrontal. Six supraoculars, with second being the largest and sixth the smallest. Four supraorbitals, with the second being the largest. The nasal is oval, the nostril positioned at its center. Postnasals absent. The tympanic cavity recessed, but the tympanum not exposed; scales surrounding the tympanum are slightly smaller than the ventrolateral scales. Two loreals, square-shaped, the first being the largest and contacting second and third supralabials. Supralabials 7-7; the first contacts the nasal, and the second largest. Infralabials 6-5, slender and elongated, with the first being the smallest. Four pairs of cervical scales, all elongated (approximately three times longer than wide), the first pair the largest. Temporals 4 (3 + 1), the dorsal largest and contacts the parietal. The lower eyelid has a distinct transparent disk (window), and both eyelids are accompanied by minute scales. The gular scales are relatively small and off-white in color. Three pairs of chin shields, the first pair in medial contact, the second pair separated by a small scale, and the third pair separated by a small scale. The mental scale is broad, occupying the anterior edge of the lower jaw; there is one larger postmental; the eyes are medium-sized, with a flat, circular transparent palpebral window in the center of the lower eyelid; the ear opening is oval, larger than the palpebral window but smaller than the eye diameter, and the anterior edge of the ear opening lacks flap-like protrusions; the tympanic membrane is recessed. The gulars number 25.

The body scales are smooth. The dorsal scales are rhomboidal, arranged in straight, imbricate rows, significantly larger than ventral and lateral scales; DBR 4 + 2 (1/2); MBSR 28. Ventral scales are diamond-shaped, arranged in straight, non-imbricate rows; PVSR 69; VSR 67. The tips of the fingers and toes (excluding claws) do not contact each other.

**Coloration.** The dorsal head and tail regions display a distinct bronze sheen, with a dark bronze patch extending between the nostrils and eyes. All dorsal head scales bear black spots. The dorsum features scattered black spots, while the regenerated tail exhibits a characteristic copper–black coloration. The anterior body and tail bear irregular dark dorsal stripes composed of melanin-rich scales, including prominent ventrolateral stripes. A continuous lateral stripe extends from the posterior orbit to the tail base. The ear opening is positioned obliquely below the eye, near the cervical junction. Ventral coloration includes a silver-gray throat and uniform reddish-brown abdomen devoid of patterning.

In preservation, the head and dorsum adopt a uniform brown hue with intensified lateral dark stripes. The dorsum and venter retain smooth integument texture, while the tail is lateral and ventral surfaces develop dark brown to black spotting. Throat and belly are bright gray, limbs are bronze, and the caudal region near the cloaca is pale gray.

### 3.4. Description of the Paratype and Variation

The paratype specimen YBU 22275 exhibits morphological similarity to the holotype across multiple diagnostic characters including SL, MBSR and DBR. A detailed comparison of the specimens is listed in Table 4.

**Etymology.** The species is named after its type locality in Heishui County, Sichuan Province, China. We suggested the common names “黑水滑蜥” in Chinese and “Heishui ground skink” in English for this species.

**Figure 7:**
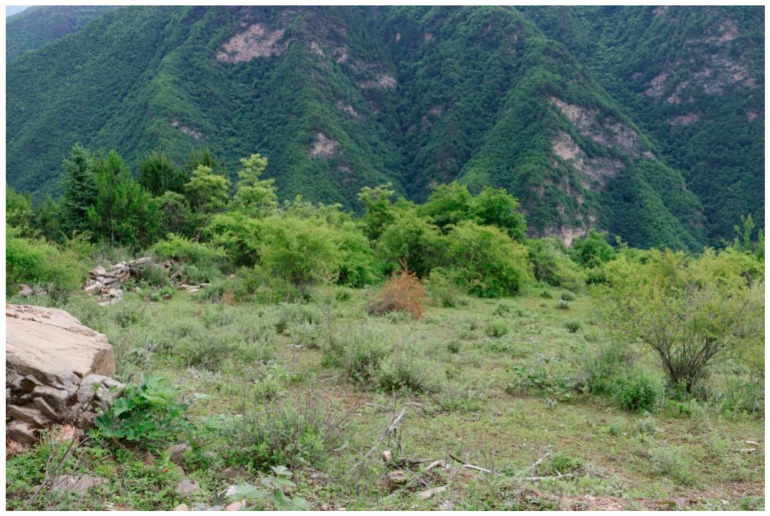
Type locality habitat of *Scincella heishuiensis* sp. nov. in Heishui County, Sichuan Province, China.

**Distribution and ecology.** Currently, *Scincella heishuiensis* **sp. nov.** has only been found in Heishui County, Sichuan Province, China. The type specimens were collected in mid-June 2022. The new species is active on rock piles in woodland areas with exposed rocks and scattered low shrubs. It is commonly found under rocks or moss on sunny days (Figure 7). This species is sympatric with *Rhabdophis nuchalis*, *Protobothrops jerdonii*, and *Gloydius swild*.

### 3.5. Comparisons with Congeners

*Scincella heishuiensis* **sp. nov**. can be distinguished morphologically from closely related homologous species in western Sichuan; the diagnostic characteristics are summarized in Table 4.

In phylogenetic trees, the new species is closely related to *S. wangyuezhaoi*, *S. modesta*, *S. tsiningensis*, and *S. huanrenensis*. Besides significant genetic divergence, the new species is morphologically distinguished from its congeners by a combination of following characters. *Scincella heishuiensis* sp. nov. can be distinguished from *S. wangyuezhaoi* and *S. modesta* by the significantly diagnostic character dorsal scale rows between dorsolateral stripes (DBR), 4 (vs. 6); and from *S. tsiningensis* and *S. huanrenensis* by fewer paravertebral scale rows (PVSR), 60–69 (vs. 70–90 in *S. tsiningensis* and 66–84 in *S. huanrenensis*). It can also be distinguished from *S. huanrenensis* by its ventral scale rows (VSR), 64–67 (vs. 52–60) [29,30].

The new species differs from *S. doriae* and *S. formosensis* by having fewer DBR (4 vs. 6). It is different from *S. monticola*, *S. qianica*, and *S. doriae* by exhibiting 28 MBSR (vs. 23–24 in *S. monticola*, 26 in *S. qianica*, 30–32 in *S. doriae*) [30,31,32,33,34]. The new species differs from *S. doriae* and *S. schmidti* by having enlarged undivided lamellae beneath toe IV 13–14 (vs. 11–12 in *S. schmidti*; 16–18 in *S. doriae*). The new species differs from *S. barbouri* in PVSR 60–69 (vs. 70–79 in *S. barbouri*). The new species can be further differentiated from *S. alia* by VSR 64–67 (vs. 44–52). *Scincella heishuiensis* **sp. nov.** can be differentiated from *S. schmidti* and *S. potanini* by fewer enlarged undivided lamellae beneath toe IV (8–10 vs. 11–12 in *S. schmidti* and 10–13 in *S. potanini*) [16,32,35,36]. The new species differs from *S. barbourin* in nuchals 2–3 (vs. 4–5). The new species can be distinguished from *S. przewalskii* by having a distinct black lateral stripe, whereas *S. przewalskii* has a pale green one. The new species has a dirty white belly, which distinguishes it from *S. przewalskii* (slate gray) and *S. tsinlingensis* (bluish gray). *Scincella heishuiensis* **sp. nov.** differs from *S. modesta* by having different anterior dorsolateral scale arrangement (straight vs. wavy in). The new species differs from *S. przewalskii* by having four supraoculars (vs. three) [37,38,39,40,41] (Table 4).

## 4. Discussion

With the description of a new cryptic species from western China, based on genetic and morphological evidence, the genus *Scincella* now comprises 51 recognized species, with 17 occurring in China. Molecular phylogenetic analyses revealed that the new species is sister to *S. wangyuezhaoi* (posterior probability [PP] = 0.78), a species recently described from Wenchuan County, Sichuan Province. The two species exhibit a genetic divergence of 5.45% (*p*-distance based on the 16S fragment). Geographically, their type localities are separated by only 100 km. Despite their proximity, they show significant genetic divergence and substantial morphological differentiation.

The Hengduan Mountains, one of the world’s youngest mountain systems, serve as a biodiversity hotspot, harboring more than half of China’s *Scincella* species, including *S. schmidti*, *S. liangshanensis*, *S. wangyuezhaoi*, *S. potanini*, *S. monticola*, *S. tsinlingensis*, *S. doriae*, and *S. reevesii* [40,41,42]. The region’s high habitat heterogeneity and riverine isolation have significantly contributed to speciation processes. As a widespread group of lizards in China, *Scincella* species exhibit complex systematic and evolutionary patterns. For instance, *S. tsinlingensis* and *S. huanrenensis* share a close phylogenetic relationship but occupy entirely distinct and geographically separated ranges. Similar challenges arise in other species within the genus, such as the *S. potanini–S. monticola* species complex. Second, there are two different reproductive models within this genus. Populations in high-altitude regions are predominantly lecithotrophic viviparity, while those in low-altitude areas are primarily oviparous [35,43]. Additionally, our research suggests the likely presence of a substantial number of cryptic species in this area [44]. Given these patterns, a key question emerges: what mechanisms drive such rich species diversity within this relatively small region? To address this, comprehensive sampling and multi-locus genetic data—particularly genomic data—are essential for unraveling the evolutionary history and speciation processes of these organisms.

## 5. Conclusions

We describe a new skink species, *Scincella heishuiensis* sp. nov., from the HDM in China, based on two specimens from San’ao Snow Mountain, Heishui County, Sichuan. The species is formally delineated using integrated morphological and genetic evidence, underscoring its distinctiveness from its congeners. This discovery not only contributes to the documented biodiversity of HDM, a globally recognized hotspot, but also suggests that the region may harbor additional cryptic diversity. The findings prompt further exploration of the drivers of *Scincella* diversification within this complex mountainous terrain and provide a scientific foundation for enhancing local conservation strategies.

## Figures and Tables

**Table 1:** Detailed information for all samples used in this study.

Species	Locality	Voucher ID	16S	12S	COI
*Scincella heishuiensis* **sp. nov.**	China: Sichuan, Heishui	YBU 22269	PX285308	PX285312	PX285297
*Scincella heishuiensis* **sp. nov.**	China: Sichuan, Heishui	YBU 22275	PX285309	PX285313	PX285298
*Scincella chengduensis*	China: Sichuan, Dayi	CIB 107637	PQ466921	PQ466924	PQ467109
*Scincella chengduensis*	China: Sichuan, Chongzhou	CIB 118786	PQ466920	PQ466923	PQ467108
*Scincella liangshanensis*	China: Sichuan, Yuexi	XM-YXS80	PP826313	PP826316	PP824805
*Scincella liangshanensis*	China: Sichuan, Meigu	CIB 119513	PP826315	PP826317	PP824806
*Scincella assata*	El Salvador: Santa Ana, Finca El Milagro	KU 289795	JF498074	JF497946	-
*Scincella badenensis*	Vietnam: Tay Ninh, Ba Den Mountain	ITBCZ 5966	-	-	MK990602
*Scincella badenensis*	Vietnam: Tay Ninh, Ba Den Mountain	ITBCZ 5993	-	-	MK990603
*Scincella baraensis*	Vietnam: Binh Phuoc, Ba Ra Mountain	ITBCZ 6534	-	-	MT742256
*Scincella baraensis*	Vietnam: Binh Phuoc, Ba Ra Mountain	ITBCZ 6536	-	-	MT742258
*Scincella boettgeri*	Japan: Southern-Ryukyu Islands, Yaeyama Group	KUZ R68001	-	-	LC630768
*Scincella boettgeri*	Japan: Southern-Ryukyu Islands, Yaeyama Group	KUZ R68008	-	-	LC630770
*Scincella cherriei*	Mexico: Chiapas, Montes Azules Biosphere Reserve	RCMX235	MW265932	-	-
*Scincella doriae*	Vietnam: Lam Dong, Bidoup-Nui Ba N. P.	ZMMU R-13268-01062	-	-	MH119617
*Scincella doriae*	Vietnam: Lam Dong, Bidoup-Nui Ba N. P.	ZMMU R-13268-00412	-	-	MH119616
*Scincella dunan*	Japan: Southern Ryukyus, Yonagunijima Is.	KUZ R65170	-	-	LC630778
*Scincella dunan*	Japan: Southern Ryukyus, Yonagunijima Is.	KUZ R67027	-	-	LC630779
*Scincella formosensis*	China: Taiwan	KUZ R37516	-	-	LC630790
*Scincella formosensis*	China: Taiwan	KUZ R37515	-	-	LC630789
*Scincella gemmingeri*	-	-	AY308294		
*Scincella lateralis*	-	KU 289460	JF498077	JF497948	-
*Scincella lateralis*	USA: Texas	DCC 2842	HM852503	HM852476	-
*Scincella melanosticta*	Vietnam: Gia Lai, Kon Chu Rang N.R.	ZMMU NAP-06376	-	-	MH119622
*Scincella melanosticta*	Vietnam: Gia Lai, Kon Chu Rang N.R.	ZMMU NAP-05519	-	-	MH119621
*Scincella fansipanensis*	Vietnam: Lao Cai Province, Fansipan	IEBR R.5185	-	-	LC846671
*Scincella fansipanensis*	Vietnam: Lao Cai Province, Fansipan	IEBR R.5187	-	-	LC846672
*Scincella modesta*	China: Zhejiang, Ningbo	WYF 11520		PP819197	PP819215
*Scincella modesta*	China: Zhejiang, Ningbo	CIB 121415	PP819195	PP819198	PP819217
*Scincella monticola*	China: Yunnan, Shangri-La	DL-YNJC2020824	OP955962	OP955952	-
*Scincella nigrofasciata*	Vietnam: Ba Ria-Vung Tau, Dinh Mountain	ITBCZ 6344	-	-	MK990605
*Scincella nigrofasciata*	Cambodia: Mondulkiri, Keo Seima W.S.	CBC 02546	-	-	MH119614
*Scincella potanini*	China: Sichuan, Kangding	DL-KD202109071	OP935937	OP942203	OP942210
*Scincella potanini*	China: Sichuan, Kangding	DL-KD202109072	OP935987	OP942208	OP942209
*Scincella vandenburghi*	Japan: Tsushima Is.	KUZ R66394	-	-	LC507695
*Scincella vandenburghi*	Korea: Yeongwol-gun	G389SV	KU646826	KU646826	KU646826
*Scincella huanrenensis*	Korea: Gangwon-do, Pyeongchang-gun	-	NC030779	NC030779	NC030779
*Scincella huanrenensis*	Korea: Gangwon-do, Pyeongchang-gun	-	KU507306	KU507306	KU507306
*Scincella wangyuezhaoi*	China: Sichuan, Wenchuan	CIB 87246	OP941172	OP942191	OQ402205
*Scincella wangyuezhaoi*	China: Sichuan, Lixian	CIB 119518	OP941173	OP942193	-
*Scincella wangyuezhaoi*	China: Sichuan, Lixian	CIB 119510	OP941174	OP942192	OQ402206
*Scincella qianica*	Baiyun District, Guizhou, China	QHU R2025001	PV527321	PV527316	PV527759
*Scincella qianica*	Baiyun District, Guizhou, China	QHU R2025002	PV527322	PV527317	PV527760
*Scincella alia*	Tay Con Linh Mt., Ha Giang, Vietnam	VRTC NAP14081	-	-	PV085567
*Scincella alia*	Tay Con Linh Mt., Ha Giang, Vietnam	ZMMU Re-18153	PV088913	PV088911	PV085569
*Sphenomorphus cryptotis*	China: Guangxi, Shangsi	CIB 119027	OP942190	OP942206	OP942215

**Table 2:** Uncorrected *p*-distances (%) between species of *Scincella* based on 16S RNA sequences.

	1	2	3	4	5	6	7	8	9	10
1. *Scincella heishuiensis* **sp. nov.**										
2. *Scincella wangyuezhaoi*	5.45									
3. *Scincella liangshanensis*	6.26	6.46								
4. *Scincella chengduensis*	5.46	5.59	2.90							
5. *Scincella assata*	8.82	8.52	7.62	7.01						
6. *Scincella potanini*	8.10	8.22	6.62	5.46	8.14					
7. *Scincella tsinlingensis*	7.88	7.80	8.48	7.68	8.82	7.69				
8. *Scincella monticola*	6.99	7.77	5.08	4.79	7.69	3.28	7.02			
9. *Scincella lateralis*	8.29	9.39	8.83	7.85	8.26	8.90	9.07	8.00		
10. *Scincella gemmingeri*	8.79	9.60	8.13	8.13	7.92	8.81	9.71	8.79	8.39	
11. *Scincella huanrenensis*	8.55	8.41	8.21	7.25	8.60	7.49	2.19	6.81	9.97	10.18

**Table 3:** Uncorrected *p*-distances (%) between species of *Scincella* based on COI sequences.

	1	2	3	4	5	6	7	8	9	10	11	12	13	14	15	16	17	18	19
1. *Scincella heishuiensis* **sp. nov.**																			
2. *Scincella alia*	16.7																		
3. *Scincella nigrofasciata*	18.3	17.6																	
4. *Scincella chengduensis*	15.6	15.3	18.7																
5. *Scincella liangshanensis*	15.3	15.6	17.5	16.1															
6. *Scincella wangyuezhaoi*	15.2	18.4	17.8	18.1	16.4														
7. *Scincella potanini*	15.6	17.4	17.1	17.0	13.7	16.4													
8. *Scincella modesta*	16.0	18.4	19.2	18.4	16.3	17.0	17.3												
9. *Scincella tsinlingensis*	17.0	19.0	19.5	17.8	16.5	18.8	18.8	19.7											
10. *Scincella vandenburghi*	16.8	17.9	18.9	16.3	16.8	17.0	16.9	15.6	19.9										
11. *Scincella boettgeri*	19.0	17.9	19.1	18.3	16.8	20.4	15.9	15.8	18.3	14.1									
12. *Scincella dunan*	18.0	19.0	19.0	18.4	19.0	19.8	18.6	16.3	18.3	15.1	7.6								
13. *Scincella formosensis*	18.2	18.4	20.0	18.3	18.7	20.2	18.7	17.1	16.9	14.4	8.8	9.6							
14. *Scincella fansipanensis*	16.4	15.7	16.2	16.7	12.5	16.4	14.5	16.4	18.4	17.7	16.8	18.7	18.8						
15. *Scincella doriae*	17.6	16.1	17.5	19.6	17.0	20.2	16.8	16.9	20.9	17.0	18.4	19.1	19.4	18.3					
16. *Scincella melanosticta*	21.3	19.0	21.0	20.0	18.6	21.2	18.9	20.7	21.3	23.1	20.5	21.9	21.2	20.1	19.1				
17. *Scincella badenensis*	18.6	18.8	10.7	19.2	17.7	18.2	18.9	20.0	19.3	21.1	19.5	19.2	20.2	16.6	19.1	19.8			
18. *Scincella baraensis*	19.2	20.8	18.7	21.8	17.6	20.8	18.3	19.9	21.6	21.2	20.8	22.4	20.6	18.7	16.8	19.0	18.9		
19. *Scincella qianica*	17.3	15.9	19.8	16.3	14.8	15.7	17.2	19.2	18.8	19.9	19.7	20.3	19.5	16.8	19.4	20.5	17.9	20.1	

**Table 4:** Diagnostic morphometric comparison between *Scincella heishuiensis* **sp. nov**. and four morphologically similar congeners from Southwest China. Character abbreviations are detailed in Section 2.

Characters	Holotype	Paratype					
	YBU 22269	YBU 22275	*Scincella heishuiensis* sp. nov.	*S. wangyuezhaoi*	*S. chengduensis*	*S. liangshanensis*	*S. potanini*
SVL	40.90	57.24	40.90–57.24	35.0–62.1	28.40–43.20	43.1–61.9	26.6–57.9
TAL	39.25	32.86	32.86–39.25	27.6–81.1	45.16–69.03	50.5–81.00	61.9–85.0
FLL	8.20	7.49	7.49–8.20	10.18–12.2	9.80–11.66	6.03–13.618	9.6–12.0
HLL	12.87	9.25	9.25–12.87	14.08–16.21	11.66–13.62	11.38–14.57	12.8–17.5
AGD	29.65	36.92	29.65–36.92	15.05–36.68	23.00–23.76	22.57–40.235	-
NU	2	3	2–3	2–4	3–3	3–3	3
SL	7–7	7–7	7–7	7–7	7–7	7–7	7–8
IfL	6–5	6–6	5–6	6–7	7–7	7–6	6–7
F4L	2.58	3.41	2.58–3.41	2.24–3.77	2.26–2.59	4.74–6.36	1.56–3.47
T4L	4.96	5.60	4.96–5.60	2.24–6.41	3.77–3.89	2.19–3.10	1.33–5.79
SC	6–5	6–7	5–7	5–7	6–7	6–7	6–7
SO	4–4	4–4	4–4	4–4	4–4	4–4	4–4
L	2–2	1–1	1–2	2–2	2–2	2–2	2–2
PN	0	2	0–2	0	0	0	0
MBSR	28	28	28	27–30	23	23–27	24–27
DBR	4	4	4	6	4	4	4
TEM	1 + 2/1 + 2	1 + 2/1 + 2	1 + 2/1 + 2	1 + 2/2 + 3	1 + 2/2	1 + 2/2 + 3	1 + 2/2 + 2
SRB	2	2	2	-	1–2	1–2	1–2
PVSR	69	60	60–69	60–75	57–60	69–80	65–84
T4S	13–13	14–13	13–14	14–15	10–12	10–15	10–13
F4S	8–8	10–10	8–10	9–11	8–9	8–11	7–10
GL	25	24	24–25	25–30	21–22	22–29	23–25
VSR	67	64	64–67	46–59	42–44	43–57	45–64
PF	2	2	2	2	2	2	2
FP	2	2	2	2	2	2	2
ADLS	straight	straight	straight	wavy	straight	straight	straight

## Data Availability

All data supporting the findings of this study are available within the article. Voucher specimens and their collection details (location, altitude, date, collector) are provided in Section 2. The newly generated mitochondrial DNA sequences (12S, 16S, COI) have been deposited in the GenBank database under accession numbers. Other data that support the findings of this study are available from the corresponding author upon reasonable request.
